# 82913 Engaging women of color virtually: Cultural Impact of Treatment Delays among Women of Color with Uterine Fibroids

**DOI:** 10.1017/cts.2021.600

**Published:** 2021-03-30

**Authors:** Minerva Orellana, Megan Allyse, Elizabeth A. Stewart, Sateria Venable, Felicity Enders, Joyce E. Balls-Berry

**Affiliations:** 1 Mayo Clinic Graduate School of Biomedical Science; 2 Mayo Clinic; 3 The Fibroid Foundation; 4 Washington University School of Medicine

## Abstract

ABSTRACT IMPACT: This study will showcase the importance if incorporating patient stakeholders in the development of an interview guide for a women of color with uterine fibroids, an understudied population. OBJECTIVES/GOALS: Black women and Hispanic/Latinas report having greater symptom burden from uterine fibroids (UF), non-cancerous neoplasms, compared to White women. These disparities may be linked to cultural factors resulting in treatment delays. The objective of this study is to provide insights to barriers and facilitators to timely treatment. METHODS/STUDY POPULATION: In partnership with the Fibroid Foundation, a UF advocacy organization, we plan to conduct a virtual community engagement (CE) studio to serve as a first step for a pilot study with a national cohort of Black women and Hispanic/Latinas who receive treatment in the United States for UF. The studios will include a presentation about UF treatment options and a facilitated discussion. The CE team will use past research and constructs from Model of Improvement and Health Belief Model to develop materials for the studio. A qualitative researcher will guide the discussion, a note-taker will take notes, and they will thematically code the notes. The results will be used to create and implement a cross-sectional in-depth qualitative study with a national sample. RESULTS/ANTICIPATED RESULTS: We hypothesize that timely treatment will be impacted by cultural factors, such as health literacy in uterine fibroids and menstruation. We expect that detailed feedback from this national cohort will contribute to greater insight to the experiences of women of color with UF and address barriers and facilitators to treatment. We anticipate the anecdotes will provide information about the influence of culture in seeking treatment for UF. We will utilize this experience to understand the impact of a virtual CE studio in elucidating open discussion among women of color on a challenging and personal topic. DISCUSSION/SIGNIFICANCE OF FINDINGS: Using CE process with advocates and research partners attains a deeper understanding in the development of an interview guide to examine the cultural impact on the treatment of UF for women of color. Understanding cultural barriers and facilitators can help overcome treatments delays in UF along with other gynecological diseases.

